# Triglyceride-Glucose Index and Homeostasis Model Assessment-Insulin Resistance in Young Adulthood and Risk of Incident Congestive Heart Failure in Midlife: The Coronary Artery Risk Development in Young Adults Study

**DOI:** 10.3389/fcvm.2022.944258

**Published:** 2022-06-30

**Authors:** Xianghui Zeng, Dunzheng Han, Haobin Zhou, Yuting Xue, Xiao Wang, Qiong Zhan, Yujia Bai, Xingfu Huang, Qingchun Zeng, Hao Zhang, Zhuang Ma, Hao Ren, Dingli Xu

**Affiliations:** ^1^Department of Cardiology, Nanfang Hospital, Southern Medical University, Guangzhou, China; ^2^Department of Rheumatology, Nanfang Hospital, Southern Medical University, Guangzhou, China

**Keywords:** triglyceride-glucose index, HOMA-IR, insulin resistance, congestive heart failure, the CARDIA study

## Abstract

**Objective:**

This study aimed to assess the association between triglyceride-glucose (TyG) index/homeostasis model assessment-insulin resistance (HOMA-IR) within young adults and congestive heart failure (CHF), and to explore whether TyG index can replace HOMA-IR as a surrogate marker for IR in predicting the risk of CHF.

**Methods:**

A total of 4,992 participants between the ages of 18 and 30 years were enrolled from the Coronary Artery Risk Development in Young Adults (CARDIA) investigation [from 1985 to 1986 (year 0)]. A Cox proportional hazard regression analysis was conducted for assessing correlations between baseline TyG index/HOMA-IR and CHF events, together with the receiver operating characteristic (ROC) curve employed for scrutinizing TyG index/HOMA-IR and the risk of CHF.

**Results:**

During the 31-year follow-up period, 64 (1.3%) of the 4,992 participants developed CHF. In multivariable Cox proportional hazards models, adjusted for confounding factors for CHF, an increased risk of CHF was associated with a per-unit increase in the TyG index [hazard ratio (HR) 2.8; 95% confidence interval (CI), 1.7–4.7] and HOMA-IR (HR 1.2; 95% CI, 1.1–1.3). A Kaplan–Meier curve analysis showed that participants in the TyG index and HOMA-IR index Q4 group had a higher risk of CHF than those in the Q1 group. The area under curve (AUC) for the TyG index and HOMA-IR consisted of 0.67 (95% CI, 0.6–0.742) and 0.675 (95% CI, 0.604–0.746), respectively. There were no significant differences between the TyG index and HOMA-IR for AUC (*p* = 0.986).

**Conclusion:**

The higher TyG index and HOMA-IR are independent risk factors for CHF. The TyG index can replace HOMA-IR in young adulthood as a surrogate marker for IR to predict the risk of CHF.

## Introduction

Congestive heart failure (CHF) is a global issue within the public-health scenario and it is also the main causative agent for mortality (1, 2). Many risk parameters, such as diabetes, hypertension, and coronary heart disease, are considered to be intimately related to heart failure (2, 3). Consequently, it is very imperative to identify any risk factors for heart failure early, followed by a timely treatment, to prevent or regulate any progress to heart failure. There is a close interplay between insulin resistance (IR) and the etiology and clinical manifestation of heart failure (4). IR is very common in patients with heart failure (5, 6). The biological effects of exacerbated IR can, in turn, lead to the development or exacerbation of heart failure (7). Multiple investigations demonstrated that IR is an independent risk factor for heart failure (8, 9). Consequently, early diagnosis of IR can predict the manifestation of future heart failure events. Methods for evaluating IR include the euglycemic-hyperinsulinemic clamp test, the quantitative insulin sensitivity check index, HOMA-IR, 1/insulin, and Matusda index (10). Although the euglycemic-hyperinsulinemic clamp test is considered to be an accurate and reliable method for the assessment of IR, such a test is time consuming, complex, and carries an increased financial running cost, rendering it a challenge for such a test to be implemented and promoted within routine clinical practice (11, 12). HOMA-IR has become the most commonly used indicator for the clinical assessment of IR (13). Triglyceride glucose (TyG) index derived from triglyceride and glucose, which was recently proposed as a reliable and inexpensive biomarker for predicting IR (as an alternative to euglycemic-hyperinsulinemic clamp test and HOMA-IR evaluation), has been used within the clinical setting and subject to focus by cardiovascular disease researchers (14). Recent investigations provided further evidence for the clinical manifestation risk of CHF, together with hypertension, ischemic stroke, arteriosclerosis, diabetes, and coronary heart disease being correlated with TyG index (15, 16). However, it is unclear whether the TyG index can predict the occurrence of heart failure. To answer this question, this study focused on the cohort from the Coronary Artery Risk Development in Young Adults (CARDIA) study to longitudinally observe whether there was an association between the TyG index/HOMA-IR and CHF. In addition, this study evaluated whether the TyG index could replace HOMA-IR as the main classifier of IR for predicting CHF event risk.

## Materials and Methods

### Study Population

From 1985 to 1986 (year 0), the CARDIA multi-center randomized, prospective cohort study was conducted, enrolling 5,115 African-American and Whites aged 18–30 years from the general population or selected census areas from four research centers in the United States. All participants were investigated at years 2, 5, 7, 10, 15, 20, 25, and 30, respectively. The institutional review committee from each research center accepted the research scheme and informed consent from all individual cohort participants was obtained in writing. The baseline data for this study used the 0-year examination data, for a total of 5,114 participants (one patient withdrew consent). Following the exclusion of patients with incomplete clinical data (missing fasting blood glucose, triglyceride, insulin, and missing endpoint records), a total of 4,992 patients formed part of the final analytical queue (Supplementary Figure 1). Patients were grouped into four groups, depending on the TyG index quartiles.

### Triglyceride-Glucose Index, Homeostasis Model Assessment-Insulin Resistance, and Congestive Heart Failure

Participants at 0 year fasted for at least 8 h, immediately followed by blood collection using an EDTA vacuum vessel. Consequently, plasma was isolated and frozen at –70°C prior to shipping to the laboratory using dry ice. Glucose was determined at baseline using hexokinase UV, calibrated, and followed by the enzymatic analysis of triglyceride levels (17). The TyG index was determined as: Ln [fasting triglycerides (mg/dl) × fasting blood glucose (mg/dl)/2] (18). HOMA-IR was determined as: fasting blood glucose (mmol/L) × fasting serum insulin (μU/ml)/22.5 (19).

Diagnostic validation of CHF necessitated a finalized CHF diagnosis by a physician, together with the implementation of CHF clinical management protocols during the patient hospitalization period (i.e., diuretic/s + digoxin/Glycerin tri-nitrate, hydralazine, ACE-inhibitor/s, or angiotensin receptor blocker/s). All patients were monitored until an endpoint of August 2017.

### Covariates

Covariates included in the present analysis were obtained through established protocols/quality assurance processes throughout all centers involved (20). The education level was stratified as high school or less and more high school. Smoking status was stratified as present and present non-smoking (such as past and never smoking). Hypertension was deemed present upon a systolic blood pressure of ≥ 130 mmHg, diastolic blood pressure of ≥ 90 mmHg, or current consumption of anti-hypertension drug/s (21). Obesity was deemed present upon a body mass index (BMI) ≥ 30 kg/m^2^ (22). The dietary modification study equation for renal disease diet was implemented in this study to estimate the glomerular filtration rate (eGFR) within serum creatinine: eGFR (ml/min/1.73 m^2^) = 175 × standardized Scr^–1^.^154^ × age^–0^.^203^ × 1.212 [if African-American] × 0.742 [if female]. Participants with eGFR < 60 ml/min/1.73 m^2^ were deemed to have chronic kidney disease (CKD) (23, 24). Detailed descriptions of measurements for total cholesterol (TC), low-density lipoprotein cholesterol (LDL-C), high-density lipoprotein cholesterol (HDL-C), triglycerides, serum creatinine, and fasting plasma glucose for all participants were previously published.

### Statistical Analyses

Normally distributed continuous data were represented by mean and standard deviation (*SD*), while non-normally distributed continuous data were represented by median with interquartile range (IQR). Categorical variables reported percentage frequency. Participants were classified into four groups according to the quartiles of the TyG index. Wilcoxon or Kruskal–Wallis test were employed for analyzing group variations for continuous variables, while the chi-square test was employed for categorical variables. Smooth curve fittings and scatter plots were used to address the relationship between the TyG index and HOMA-IR. The Cox proportional-hazard regression model was employed to determine hazard ratio (HR) and 95% CI for CHF events by quartiles of the TyG index, and HOMA-IR, respectively. The proportional hazard assumption was evaluated by the visualization of Schoenfeld residuals, where such analytical outcomes indicated no evidence of assumption breaches (Supplementary Table 1). Multi-collinearity was investigated using variance inflation factors, while TC was removed as a significant variance inflation factor (≥5). Three models were fitted: model 1 was not adjusted; model 2 was adjusted for age, sex, and race; and model 3 was adjusted for variables included in the model 2 and education level, smoking status, hypertension, diabetes mellitus, hypercholesteremia, systolic and diastolic blood pressure, obesity, CKD, HDL-C, and LDL-C. Trend *p*-values were evaluated by a median value within each quartile, as a continuous variable. Kaplan–Meier curve data outcomes were employed for determining the cumulative incidence of CHF events through both the TyG index and HOMA-IR quartiles, with estimation variations being comparatively analyzed through log-rank protocols. The receiver operating characteristic (ROC) curve and area under the curve (AUC) were used to assess both the TyG index-based and HOMA-IR-based capacity for predicting CHF event risk during follow-up. The participants were divided into subgroups according to sex, race, education, obesity, smoking status, hypertension, and CKD status. The results were scrutinized following adjustments for age, sex, race, education, obesity, smoking status, hypertension, diabetes mellitus, hypercholesteremia, CKD, LDL-C, and HDL-C, except for the subgroup variable. All statistical analyses were conducted using R^®^ software (version 4.0.3).^1^ The study deemed that *p*-values less than 0.05 (bilateral) conferred statistical significance.

## Results

Table 1 shows the baseline characteristics for the total-participating patient cohort, together with the quartile TyG index. During a median (IQR) follow-up of 31 (30.8–31.2) years, 64 out of 4,992 participants (1.3%) developed CHF, with an annual incidence of 41.4/100,000 individuals. With the increase in the quartile of the TyG index of participants, the CHF events increased significantly, from 20.7/100,000 individuals in quartile Q1 to 82.7/100,000 in quartile Q4. The median (IQR) of the TyG index quartiles were 7.3 (7.1–7.4), 7.7 (7.6–7.8), 8.0 (7.9–8.1), and 8.4 (8.3–8.7), respectively. With decreasing quartiles of TyG index, the prevalence of obesity, hypercholesteremia, hypertension, and current smoking were progressively higher (all *p* < 0.001), paralleling the progressive increase of triglycerides, systolic and diastolic blood pressure, HOMA-IR, insulin, HDL-C, LDL-C, TC, and fasting blood glucose (all *p* < 0.001). Conversely, the proportion of women and Whites was progressively lower with the increasing quartiles of the TyG index (all *p* < 0.001).

**TABLE 1:** Clinical characteristics of the study population according to the TyG index.

Variables	Overall	Quartiles of TyG index				*P*-value
		Q1	Q2	Q3	Q4	
No. of participants	4,992	1,247	1,246	1,251	1,248	
TyG index	7.8 (7.5–8.2)	7.3 (7.1–7.4)	7.7 (7.6–7.8)	8.0 (7.9–8.1)	8.4 (8.3–8.7)	< 0.001
Age, year	25.0 (22.0–28.0)	25.0 (22.0–28.0)	25.0 (22.0–28.0)	25.0 (22.0–28.0)	26.0 (23.0–28.0)	<0.001
Female, *n* (%)	2721 (54.5%)	807 (64.7%)	737 (59.1%)	676 (54.0%)	501 (40.1%)	<0.001
White, *n* (%)	2562 (51.3%)	754 (60.5%)	680 (54.6%)	618 (49.4%)	510 (40.9%)	<0.001
More high school, *n* (%)	2989 (60.0%)	745 (59.9%)	747 (60.0%)	765 (61.2%)	732 (58.8%)	0.681
Obesity, *n* (%)	578 (11.6%)	77 (6.2%)	105 (8.5%)	148 (11.8%)	248 (20.0%)	<0.001
Systolic BP, mmHg	110.0 (103.0–118.0)	107.0 (101.0–115.0)	108.0 (102.0–116.0)	110.0 (104.0–118.0)	113.0 (106.0–121.0)	<0.001
Diastolic BP, mmHg	68.0 (62.0–75.0)	67.0 (62.0–72.0)	67.0 (62.0–74.0)	69.0 (63.0–75.0)	70.0 (64.0–77.0)	<0.001
Current smoking, *n* (%)	1521 (30.5%)	296 (23.8%)	387 (31.1%)	378 (30.2%)	460 (36.9%)	<0.001
Diabetes mellitus, *n* (%)	37 (0.7%)	7 (0.6%)	6 (0.5%)	6 (0.5%)	18 (1.5%)	<0.001
Hypertension, *n* (%)	975 (19.5%)	182 (14.6%)	185 (14.8%)	263 (21.0%)	345 (27.6%)	<0.001
Hypercholesteremia, *n* (%)	102 (2.1%)	16 (1.3%)	15 (1.2%)	24 (2.0%)	47 (3.9%)	<0.001
CKD, *n* (%)	159 (3.2%)	34 (2.7%)	35 (2.8%)	42 (3.4%)	48 (3.8%)	0.351
HDL-C, mg/dL	52.0 (44.0–61.0)	56.0 (50.0–66.0)	54.0 (47.0–63.0)	51.0 (44.0–59.0)	44.0 (37.0–53.0)	<0.001
TC, mg/dL	174.0 (153.0–197.0)	163.0 (144.0–182.0)	169.0 (150.0–189.8)	176.0 (157.0–200.0)	188.0 (167.0–214.0)	<0.001
LDL-C, mg/dL	106.0 (87.0–127.0)	97.0 (80.0–115.0)	102.0 (85.0–121.0)	110.0 (91.0–131.5)	119.0 (97.0–140.0)	<0.001
Triglycerides, mg/dL	62.0 (45.0–84.0)	38.0 (32.5–42.0)	53.0 (49.0–58.0)	71.0 (66.0–78.0)	108.0 (93.0–140.0)	<0.001
Fasting glucose, mg/dL	81.0 (77.0–87.0)	78.0 (74.0–83.0)	81.0 (76.0–85.0)	82.0 (78.0–87.0)	85.0 (80.0–91.0)	<0.001
HOMA-IR	1.8 (1.2–2.7)	1.4 (1.0–2.0)	1.6 (1.1–2.4)	1.9 (1.3–2.7)	2.4 (1.5–3.6)	<0.001
Insulin, μU/mL	8.8 (6.1–13.0)	7.3 (5.1–10.2)	8.1 (5.8–11.6)	9.3 (6.4–13.5)	11.2 (7.4–16.9)	<0.001
Follow-up time, years	31.0 (30.8–31.2)	31.0 (30.8–31.2)	31.0 (30.8–31.2)	31.0 (30.8–31.2)	31.0 (30.8–31.2)	<0.001
CHF	64 (1.3%)	8 (0.6%)	10 (0.8%)	14 (1.1%)	32 (2.6%)	<0.001
Incidence rate per 100,000	41.4	20.7	25.9	36.1	82.7	<0.001

*Values are presented as Median (interquartile range, IQR) or number (%). Abbreviations: BMI, body mass index; Systolic BP, systolic blood pressure; Diastolic BP, diastolic blood pressure; HDL-C, high-density lipoprotein cholesterol; LDL-C, low-density lipoprotein cholesterol; TyG, Triglyceride-glucose; TC, total cholesterol; CKD, chronic kidney diseases; HOMA-IR, homeostasis model assessment-insulin resistance; CHF, congestive heart failure.*

**FIGURE 1:**
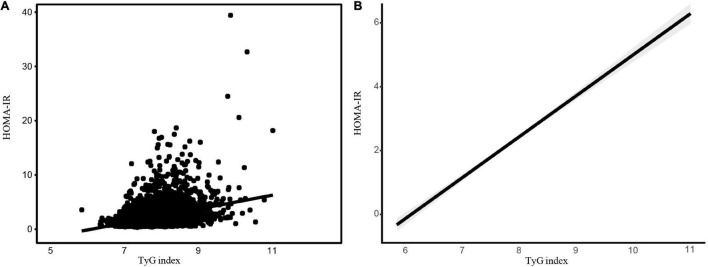
The association between the TyG index and HOMA-IR. The association between the TyG index and HOMA-IR. **(A)** Each black point represents a sample. **(B)** A solid black line represents the smooth curve fit between variables. The gray area represents the 95% of confidence interval (CI) from the fit. Abbreviations: TyG, Triglyceride-glucose; HOMA-IR, homeostasis model assessment-insulin resistance.

The scatter plots and smooth curve fittings in Figure 1 demonstrated the relationship between TyG index and HOMA-IR, where the TyG index was intimately linked to HOMA-IR (*R* = 0.339, *p* < 0.001).

**FIGURE 2:**
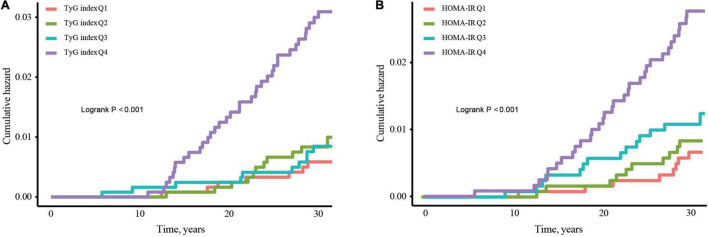
Kaplan–Meier curves for incidence of CHF stratified by quartiles of TyG index **(A)** and HOMA-IR **(B)**. Abbreviations: TyG, Triglyceride-glucose; HOMA-IR, homeostasis model assessment-insulin resistance; CHF, congestive heart failure.

On completing risk analysis, the cumulative CHF incidence among the TyG index and HOMA-IR quartile are illustrated in Figure 2. Participants in the quartile of the TyG index Q4 were at an increased risk of CHF events in comparison to the Q1 group throughout the clinical monitoring timeframe (log-rank test, *p* < 0.001; Figure 2A). Similar results were observed for HOMA-IR (Figure 2B).

The risk of CHF events was increased, with a per-unit increase in the TyG index and HOMA-IR illustrated in Table 2. In the non-adjusted model (model 1), per-unit increase in the TyG index correlated to a threefold increase for the risk of CHF (HR 3.0, 95% CI 2.1–4.4). In model 2, adjusted according to race, sex, and age, per-unit increase in the TyG index increased the risk of CHF by a 3.4-fold (HR 3.4, 95% CI 2.3–5.0). In model 3, the risk of CHF event with a per-unit increase in the TyG index was still considerable post-modification for possible confounding factors, with the HR being 2.8-fold (HR 2.8, 95% CI 1.7–4.7). The values of HR for HOMA-IR Models 1, 2, and 3 were 1.2, 1.2, and 1.2, respectively (*p* < 0.001). The risk of CHF events was still significant, based on the TyG index and the quartile of HOMA-IR (*p*-trend < 0.001).

**TABLE 2:** Hazard ratio (HR) and 95% confidence intervals (CIs) for CHF according to the TyG index and HOMA-IR.

Variables	HR (95% CI)		
	Model 1	Model 2	Model 3
**Quartiles of TyG index**			
Q1	1 (reference)	1 (reference)	1 (reference)
Q2	1.3 (0.5, 3.2)	1.3(0.51, 3.3)	1.2 (0.5, 3.1)
Q3	1.8 (0.8, 4.3)	2.0 (0.8, 4.6)	1.7 (0.7, 4.1)
Q4	4.2 (2.0, 9.1)	4.8 (2.2, 10.6)	3.4 (1.4, 8.0)
*p*-trend	<0.001	<0.001	<0.001
TyG index	3.0 (2.1, 4.4)	3.4 (2.3, 5.0)	2.8 (1.7, 4.7)
**Quartiles of HOMA-IR**			
Q1	1 (reference)	1 (reference)	1 (reference)
Q2	1.6 (0.6, 4.1)	1.7 (0.6, 4.3)	1.7 (0.6, 4.3)
Q3	1.4 (0.6, 3.8)	1.6 (0.6, 4.0)	1.3 (0.5, 3.5)
Q4	5.3 (2.4, 11.9)	4.4 (2.0, 10.0)	3.2 (1.3, 7.9)
*p*-trend	<0.001	<0.001	<0.001
HOMA-IR	1.2 (1.1, 1.2)	1.2 (1.1, 1.2)	1.2(1.1, 1.3)

*Model 1 does not adjust covariates. Model 2: adjusted for age, sex and race. Model 3: model 2 + adjusted for education, obesity, smoking status, hypertension, diabetes mellitus, hypercholesteremia, CKD, LDL-C and HDL-C. Abbreviations: HDL-C, high-density lipoprotein cholesterol; LDL-C, low-density lipoprotein cholesterol; TyG, Triglyceride-glucose; CKD, chronic kidney diseases; HOMA-IR, homeostasis model assessment-insulin resistance; CHF, chronic heart failure.*

**FIGURE 3:**
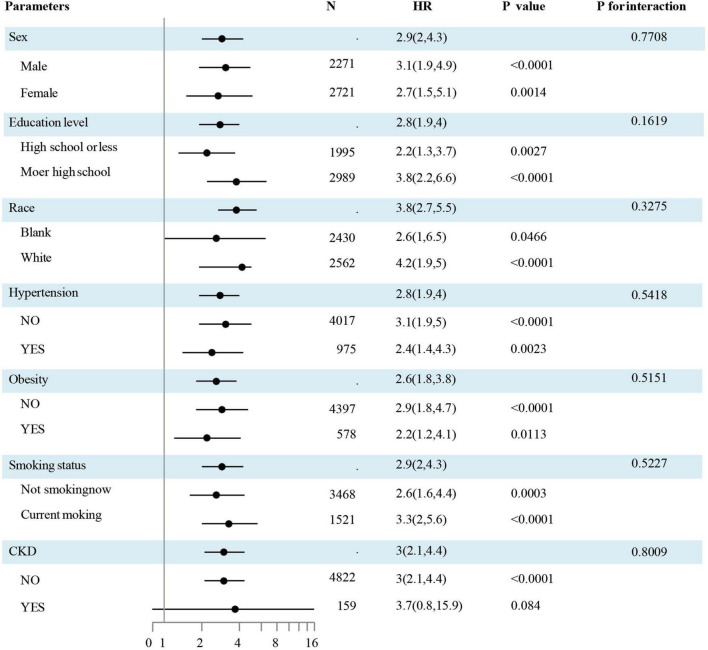
The association of CHF and the TyG index by a subgroup analysis. The association of CHF and the TyG index by a sub-group analysis. Data are hazard ratios (HRs) and 95% confidence limits (95% CLs). The participants were divided into subgroups according to sex, race, education, obesity, smoking status, hypertension, and CKD. The results were evaluated after adjusted for age, sex, race, education, obesity, smoking status, hypertension, diabetes mellitus, hypercholesteremia, CKD, LDL-C, and HDL-C except for the sub-group variable. Abbreviations: HDL-C, high-density lipoprotein cholesterol; LDL-C, low-density lipoprotein cholesterol; TyG, Triglyceride-glucose; CKD, chronic kidney diseases; HOMA-IR, homeostasis model assessment-insulin resistance; CHF, congestive heart failure.

Data outcomes from subgroup evaluations are illustrated in Figure 3. No significant interactions by sub-groups were observed for the association between sex and race, education level, obesity, smoking status, hypertension, diabetes mellitus, hypercholesteremia, or CKD status.

**FIGURE 4:**
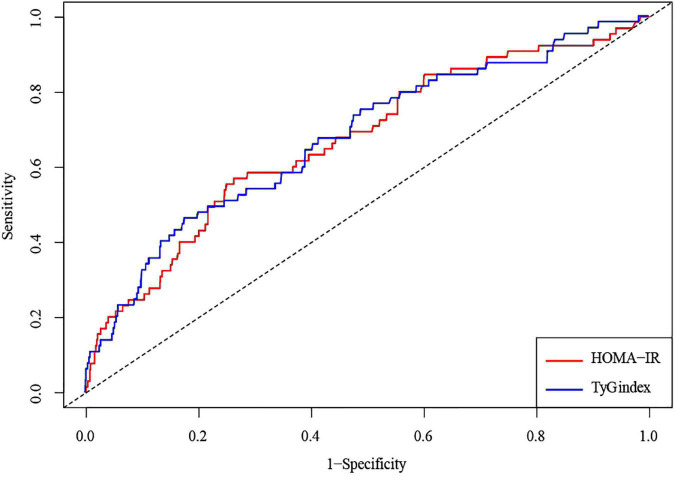
An ROC analysis of the TyG index and HOMA-IR to predict the incident risk of CHF. A solid blue line represents the TyG index; a solid red line represents HOMA-IR. Abbreviations: ROC, receiver operating characteristic; AUC, area under the curve; TyG, Triglyceride-glucose; HOMA-IR, homeostasis model assessment-insulin resistance; CHF, congestive heart failure.

The AUCs of the TyG index and HOMA-IR to predict CHF incidence were 0.675 (95% CI, 0.604–0.746) and 0.67 (95% CI, 0.6–0.742), respectively. However, such data outcomes did not exhibit significant variations (*p* = 0.986) (Figure 4 and Table 3).

**TABLE 3:** The area under the curve (AUC) of the TyG index and HOMA-IR to predict CHF incidence.

Variables	AUC (95%CI)	*P*-value*
HOMA-IR	0.670 (0.600–0.742)	Reference
TyG index	0.675 (0.604–0.746)	0.986

*Asterisk compared with HOMA-IR. Abbreviations: TyG, Triglyceride-glucose; HOMA-IR, homeostasis model assessment-insulin resistance; CHF, congestive heart failure.*

## Discussion

The main results of this prospective observational cohort study of 4,992 young Americans revealed that the TyG index in young adulthood was positively correlated with the incidence of CHF, both pre-and post-adjustments for confounders, and this correlation remained stable even on subgroup analyses, rendering the TyG index to be a potential independent risk factor for CHF. In addition, this investigation validated HOMA-IR as a separate independent risk factor for CHF, in agreement with results previously described in scientific literature. Finally, the study analyzed the AUC of CHF incidence based on the TyG index and HOMA-IR. Through comparative analyses, the TyG index shares the same predicative value as HOMA-IR in predicting CHF incidence. TyG index can be employed as a surrogate marker for IR to predict CHF incidence. The results of this extended, prospective, observational investigation have substantial weight in aiding CHF prophylaxis.

It is well acknowledged that IR is intimately linked to the development of heart failure. IR was first identified to be separately linked with the risk of heart failure following the Uppsala longitudinal study of adult men over the age of 70 years (25). In addition, it was reported that IR was capable of predicting the occurrence risk of ventricular systolic and diastolic dysfunction within 20 years among men in their 50s (26, 27). The analyses of IR require complex methods that are challenging to obtain during routine clinical practice (28). HOMA-IR is commonly used for testing IR (13). However, there is no routine measurement of insulin concentration in clinical practice, which leads to HOMA-IR being unsuitable for large-scale clinical implementation. The TgY index is indicative of the metabolic level of triglycerides and glucose, which was first proposed by Simental-Mendía et al. stating that the TyG index can replace the euglycemic-hyper-insulinemic clamp test and HOMA-IR to evaluate IR in healthy participants (18, 29). An increased TyG index is associated with the occurrence of CHF, possibly since IR is recognized as a pivotal player in abnormal glucolipid metabolism (30). Under IR, insulin-mediated glucose uptake in myocytes and adipocytes is impaired, the inhibition against liver glucose production and lipolysis is weakened, while the levels of plasma glucose and triglycerides are increased (31, 32). The increase in blood glucose levels can cause myocardial fibrosis, increased stiffness, and myocardial remodeling, typically leading to the occurrence and development of heart failure (1). Previous studies have also confirmed a positive correlation between the increase in triglycerides and the development of heart failure (33).

Meanwhile, the TyG index is correlated with various risk factors for heart failure. In a 9-year follow-up cohort study on hypertension in China, the TyG index predicted the incidence of hypertension (34). Acute coronary syndrome, hypertension, diabetes mellitus, and other factors can cause cardiac function and structural disorders, leading to heart failure (35, 36). A recent investigation on 546 patients with CHF and type II diabetes mellitus discovered a higher rate of heart failure re-hospitalization and cardiovascular mortality with the TyG index of 9.06, when compared with the TyG index of 8.55 (37). In a study of patients undergoing echocardiography at a hospital in southern Taiwan Province, China, Chiu et al. highlighted a high TyG index to be associated with an increased left atrial diameter and a reduced left ventricular ejection fraction (38). Furthermore, this investigation revealed the predictive value of HOMA-IR on CHF. The increased risk of CHF in the high HOMA-IR population has already been demonstrated in previous studies on patients with diabetes mellitus combined with chronic renal disease, though without coronary heart disease (39). However, this study demonstrated that if individuals are presented with high HOMA-IR despite being young and healthy, their risks of CHF in the future are also increased. The same finding was discovered in the long-term follow-up study of 15,792 cases (aged—45–64 years) by Vardeny et al., stating that HOMA-IR is an independent predictor of heart failure (9). According to Kishi et al., increased IR in young people is an important life-long risk of left ventricular re-modeling and dysfunction in adulthood (40).

This study also demonstrated that the TyG index and HOMA-IR had similar predictive powers for CHF events, with AUC values [0.675 (95% CI, 0.604–0.746) vs. 0.67 (95% CI, 0.6–0.742) *p* = 0.986]. HOMA-IR was employed to assess the relationship between IR and disease (41). However, the TyG index in clinical practice is simpler to perform, rather than HOMA-IR detection, and is cheaper and easier to obtain. Therefore, the TyG index has added advantages in comparison to HOMA-IR regarding the clinical evaluation and prediction of CHF. The TyG index can be used as an alternative index to predict heart failure events.

We are aware of several limitations in our study. The CARDIA study recruited only young people at the beginning of the research and did not consider people of differing ages and constitutions. Moreover, CARDIA data analysis by ethnicity was limited to African-American and white-American adult individuals, and therefore, such results cannot be cautious to other ethnic groups. Future studies are needed to assess the prevalence of CHF in other ethnicities as well as in children, athletes, and in individuals with specific diseases. Finally, the study did not compare the euglycemic-hyperinsulinemic clamp test (the gold standard for measuring IR) with the TyG index.

## Conclusion

This study suggests that the TyG index and HOMA-IR in young adulthood are independent risk factors for the development of CHF. However, the TyG index can be easily popularized in clinical practice by low-cost experimental analyses. Heart failure is a major cause of global mortality, resulting in serious economic and social burden. Early identification and intervention of people with an increased TyG index can reduce the incidence of CHF. In view of the increasing prevalence of abnormal glucose and lipid metabolism and high IR, these findings are of great significance to public health.

## Data Availability Statement

The original contributions presented in this study are included in the article/Supplementary Material, further inquiries can be directed to the corresponding author/s.

## Ethics Statement

Ethical review and approval was not required for the study on human participants in accordance with the local legislation and institutional requirements. The patients/participants provided their written informed consent to participate in this study.

## Author Contributions

XZ and DH performed data analysis and wrote the manuscript. HBZ, XW, and YX contributed to the analysis plan and reviewed and edited the manuscript. QZ, YB, XH, HZ, ZM, and QCZ contributed to the discussion. DX and HR had full access to all of the data in the study, reviewed, edited the manuscript, and took responsibility for the integrity of the data and the accuracy of the data analysis. All authors contributed to the article and approved the submitted version.

## Conflict of Interest

The authors declare that the research was conducted in the absence of any commercial or financial relationships that could be construed as a potential conflict of interest.

## Publisher’s Note

All claims expressed in this article are solely those of the authors and do not necessarily represent those of their affiliated organizations, or those of the publisher, the editors and the reviewers. Any product that may be evaluated in this article, or claim that may be made by its manufacturer, is not guaranteed or endorsed by the publisher.

## Footnotes

1
http://www.R-project.org/

## Abbreviations

CARDIA: Coronary Artery Risk Development in Young Adults
TyG: Triglyceride-glucose
HOMA-IR: Homeostasis model assessment-insulin resistance
CHF: Congestive heart failure
ROC: Receiver operating characteristic
HR: Hazard ratio
CI: Confidence interval
AUC: Area under the curve
IR: Insulin resistance
BMI: Body mass index
eGFR: Estimate the glomerular filtration rate
CKD: Chronic kidney disease
LDL-C: Low-density lipoprotein cholesterol
HDL-C: High-density lipoprotein cholesterol
TC: Total cholesterol.
