# Supplementary material for: Triglyceride-Glucose Index and Homeostasis Model Assessment-Insulin Resistance in Young Adulthood and Risk of Incident Congestive Heart Failure in Midlife: The Coronary Artery Risk Development in Young Adults Study
Source: Front Cardiovasc Med. 2022 Jun 30;9:944258. doi: 10.3389/fcvm.2022.944258 (PMC9279654; doi:10.3389/fcvm.2022.944258)
Supplement: Supplementary file 1 [file Table_1.DOCX]

Supplement

Table S1. Proportionality assumptions test for primary exposure variables

| **Variables** | **Wald χ2** | **P value** |
| --- | --- | --- |
| TyG index | 0.1559 | 0.6930 |
| HOMA-IR | 0.0481 | 0.8263 |

Abbreviation: TyG, triglyceride-glucose; HOMA-IR, Homeostasis Model Assessment-Insulin Resistance.

Participants were enolled at 0 year

(N=5114)

-Missing baseline data of fasting blood glucose(N=82), triglyceride(N=51) and insulin(N=71) in Year 0

-Missing follow-up endpoint records(N=1)

Baseline and follow-up endpoint records data available (N=4992)

Supplementary Figure S1. Flowchart of the study
